# Isolation and Pharmacological Characterization of α-Elapitoxin-Oh3a, a Long-Chain Post-Synaptic Neurotoxin From King Cobra (*Ophiophagus hannah*) Venom

**DOI:** 10.3389/fphar.2022.815069

**Published:** 2022-03-07

**Authors:** Tam M. Huynh, Anjana Silva, Geoffrey K. Isbister, Wayne C. Hodgson

**Affiliations:** ^1^ Monash Venom Group, Department of Pharmacology, Biomedical Discovery Institute, Monash University, Clayton, VIC, Australia; ^2^ Department of Parasitology, Faculty of Medicine and Allied Sciences, Rajarata University of Sri Lanka, Anuradhapura, Sri Lanka; ^3^ Clinical Toxicology Research Group, University of Newcastle, Newcastle, NSW, Australia

**Keywords:** *Ophiophagus hannah*, snake, antivenom, neuromuscular paralysis, neurotoxin

## Abstract

The King Cobra (*Ophiophagus hannah*) is the world’s largest venomous snake and has a widespread geographical distribution throughout Southeast Asia. Despite proteomic studies indicating the presence of postsynaptic neurotoxins in *O. hannah* venom, there are few pharmacological investigations of these toxins. We isolated and characterized α-elapitoxin-Oh3a (α-EPTX-Oh3a; 7,938 Da), a long-chain postsynaptic neurotoxin, which constitutes 5% of *O. hannah* venom. α-EPTX-Oh3a (100–300 nM) caused concentration-dependent inhibition of indirect twitches and inhibited contractile responses of tissues to exogenous acetylcholine and carbachol, in the chick biventer cervicis nerve-muscle preparation. The prior incubation of tissues with Thai Red Cross Society King Cobra antivenom (1 ml/0.8 mg) prevented the *in vitro* neurotoxic effects of α-EPTX-Oh3a (100 nM). The addition of Thai Red Cross Society King Cobra antivenom (1 ml/0.8 mg), at the t_90_ time point partially reversed the *in vitro* neurotoxicity of α-EPTX-Oh3a (100 nM). Repeatedly washing the tissue did not allow significant recovery from the *in vitro* neurotoxic effects of α-EPTX-Oh3a (100 nM). α-EPTX-Oh3a demonstrated pseudo-irreversible antagonism of concentration-response curves to carbachol, with a pA_2_ of 8.99. *De novo* sequencing of α-EPTX-Oh3a showed a long-chain postsynaptic neurotoxin with 72 amino acids, sharing 100% sequence identity with Long neurotoxin OH-55. In conclusion, the antivenom is useful for reversing the clinically important long-chain α-neurotoxin-mediated neuromuscular paralysis.

## Introduction

The King Cobra (*Ophiophagus hannah*) is widely distributed in Southeast Asia, some parts of the Indian subcontinent and Southern China (O'Shea, 2008). Despite typically avoiding human habitat, *O. hannah* is considered to be a medically important species since, as the world’s largest venomous snake, it is able to inject a large amount of venom in a single bite (Gowtham et al., 2012). Envenoming by *O. hannah* causes life-threatening neuromuscular paralysis and is usually treated by administration of monovalent Thai Red Cross Society King Cobra antivenom (Tin et al., 1991; Tun et al., 1995).

Few pharmacological studies have investigated the mechanism of action of *O. hannah* venom, and isolated neurotoxins, at the skeletal neuromuscular junction. Snake venom neurotoxins are generally classified by their target site at the neuromuscular junction (i.e., post-synaptic or pre-synaptic). Proteomic studies of the venom of *O. hannah* have indicated a large relative abundance of “postsynaptic” neurotoxins (Petras et al., 2015; Tan et al., 2015), which inhibit neurotransmission by acting as antagonists at the skeletal nicotinic acetylcholine receptor (nAChR) (Barber et al., 2013). Postsynaptic neurotoxins are further classified as short-chain or long-chain neurotoxins, and their structural and functional differences have been previously described in detail (Barber et al., 2013; Utkin, 2019). Recent research has shown that long-chain α-neurotoxins are more clinically important in human envenoming, compared to short-chain α-neurotoxins, due to their higher potency and poor reversibility on the human nAChR (Silva et al., 2018).

Although several α-neurotoxins have been previously isolated from *O. hannah* venom to study their protein structure (Lin et al., 1995a; Lin et al., 1995b; Chang et al., 1995; Lin et al., 1997; Chang et al., 2000; Roy et al., 2010), there is a need for pharmacological studies to characterize the main neurotoxins that potentially contribute to human neuromuscular paralysis. This study aimed to isolate the main neurotoxins from *O. hannah* venom and identify the mode of action, as well as determine the *in vitro* efficacy of Thai Red Cross Society King Cobra antivenom in neutralizing them.

## Materials and Methods

### Venoms and Antivenoms

Freeze-dried *O. hannah* venom from Indonesia was a gift from Venom Supplies Tanunda (South Australia). Thai Red Cross Society King Cobra antivenom was purchased from Thai Red Cross Society (Bangkok, Thailand; Batch No: LH00118, expiry date: 13/02/2023). According to the manufacturer’s instructions, 1 ml of the antivenom neutralizes 0.8 mg of *O. hannah* venom. The amount of antivenom required for neutralizing the *in vitro* neurotoxicity of the isolated toxin was calculated based on the relative abundance of the toxin in the whole venom.

### Chemicals and Reagents

The following chemicals and drugs were used: acetylcholine chloride (Sigma-Aldrich, St. Louis, MO, United States), acetic acid sodium salt (sodium acetate, Merck, Darmstadt, Germany), acetonitrile (ACN, Merck, Darmstadt, Germany), ammonium bicarbonate (Sigma-Aldrich, St. Louis, MO, United States), carbamylcholine chloride (carbachol; Sigma-Aldrich, St. Louis, MO, United States), dithiothretiol (Merck, Darmstadt, Germany), formic acid (Sigma-Aldrich, St. Louis, MO, United States), iodoacetamide (GE Healthcare, Uppsala, Sweden), LCMS grade acetonitrile (Fisher Scientific, Loughborough, United Kingdom), potassium chloride (KCl, Ajax Finechem Pty. Ltd., Taren Point, Australia), proteomics grade bovine trypsin (Sigma-Aldrich, St. Louis, MO, United States), trifluoroacetic acid (TFA, Auspep, Melbourne, Australia), d-tubocurarine (Sigma-Aldrich, St. Louis, MO, United States) and trifluroethanol (Sigma-Aldrich, St. Louis, MO, United States). Unless otherwise indicated, all chemicals were dissolved or diluted in milli-Q water.

### Isolation and Purification of Toxin

Chromatography was performed using a high-performance liquid chromatography (HPLC) system (Shimadzu, Kyoto, Japan).

#### Reverse-Phase HPLC

Freeze-dried *O. hannah* venom (2 mg) was reconstituted in 500 μl milli-Q water (Millipore Corporation, Billerica, MA, United States) and centrifuged at 12,000 rpm for 10 min before being loaded into a Phenomenex Jupiter semi-preparative C18 column (5 µm, 250 mm × 10 mm; Phenomenex, Torrance, CA, United States) equilibrated with solvent A (0.1% TFA) at a flow rate of 2 ml/min. The fractions were then eluted using a gradient of solvent B (90% ACN in 0.09% TFA); 0–25% over 10 min, 25%–80% between 10 and 60 min and 80–0% between 60 and 65 min. The eluent was monitored at 214 nm. Fractions were collected manually according to the peaks in the chromatogram and freeze-dried immediately, then later screened using the chick biventer cervicis nerve-muscle preparation to identify those with neurotoxicity.

#### Ion-Exchange Chromatography

Freeze-dried, single run sample of the peak eluting at 19 min from reverse-phase HPLC was reconstituted in 500 μl of buffer A (5 mM sodium acetate; pH 5.1) and loaded into a Mono S strong cation exchange column (8 μm, 8 mm × 75 mm; Showa Denko; Japan). The column was equilibrated with buffer A at a flow rate of 0.5 ml/min. The fractions were eluted using the following gradient of buffer B (5 mM sodium acetate and 0.5 M sodium chloride; pH 5.1): 0% over 10 min, 0–60% between 10 and 70 min and 60–0% between 70 and 75 min. The eluent was monitored at 280 nm. Toxin 1C was collected manually at 52 min and then subsequently freeze-dried to be ready for further analysis.

### Mass Spectrometry and Amino Acid Sequencing

#### Intact Protein Analysis With Matrix Associated Laser Desorption Time of Flight (MALDI-TOF) Mass Spectrometry

Venom fractions were analysed by LC-MS using a quadrupole TOF mass spectrometer (MicroTOFq, Bruker Daltonics, Bremen, Germany) coupled online with a 1,200 series capillary HPLC (Agilent Technologies, Santa Clara, CA, United States). Samples were injected onto a MabPac SEC-1 5um 300A 50 × 4 mm (Thermo Scientific) column with 50% acetonitrile 0.05% TFA, 0.05% FA at a flow rate of 50 μl/min. The protein was eluted over a 20 min run-time monitored by UV detection at 254 nm. The eluant was nebulised and ionised using the Bruker electrospray source with a capillary voltage of 4500 V dry gas at 180°C, a flow rate of 4 l/min and nebuliser gas pressure at 300 mbar. After 20 min, the flow path was switched to infuse Low concentration Tune mix (Agilent Technologies, Santa Clara, CA, United States) to calibrate the spectrum post-acquisition. The spectra were extracted and deconvoluted using Data explorer software version 3.4 build 192 (Bruker Daltonics, Bremen, Germany).

#### Electrospray-Ionisation Coupled With Mass-Spectrometry/Mass Spectrometry (ESI-LCMS/MS)

The sample (2.5–5 µg) was buffer exchanged into 50 mM ammonium bicarbonate and reduced in 2.5 mM DTT at 60°C for 5 min followed by alkylation with 10 mM chloroacetamide for 30 min at room temperature. The enzyme was then added at the rate of 0.5 μg per 10 μg of protein and incubated at 37°C overnight. For chymotrypsin digests 50 mM Tris buffer was used in place of ammonium bicarbonate.

All enzyme digests were analysed by LC-MS/MS using the QExactive mass spectrometer (Thermo Scientific, Bremen, Germany) coupled online with a RSLC nano HPLC (Ultimate 3,000, Thermo Scientific, Bremen, Germany). Sample (200 ng) was injected and concentrated on a 100 μm, 2 cm nanoviper pepmap100 trap column with 97.5% buffer A (0.1% TFA) at a flow rate of 15 μl/min. The peptides then eluted and separated with a Thermo RSLC pepmap100, 75 μm × 50 cm, 100 Ǻ pore size, reversed phase nano column with a 30 min gradient of 92.5% buffer A (0.1% formic acid) to 42.5% B (80% ACN 0.1% formic acid), at a flow rate of 250 nl/min. The eluant was nebulised and ionised using the Thermo nano electrospray source with a distal coated fused silica emitter (New Objective, Woburn, MA, United States) with a capillary voltage of 1900 V. Peptides were selected for MSMS analysis in Full MS/dd-MS2 (TopN) mode with the following parameter settings: TopN 10, resolution 70,000, MSMS AGC target 5e5, 118 ms Max IT, NCE 27, 1.8 m/z isolation window, dynamic exclusion was set to 10 s.

#### De Novo Protein Sequencing

Data from the LCMS/MS acquisitions was analysed using Peaks AB Ver. 2.0 (Bioinformatics Solutions Inc., Ont, Canada), to derive a *de novo* protein sequence. The following search parameters were used: missed cleavages, 2; peptide mass tolerance, ± 10 ppm; peptide fragment tolerance, ± 0.05 Da; fixed modifications, carbamidomethyl (Cys); Variable modification, oxidation (Met).

Sequences were validated using Byonic (ProteinMetrics) V 3.1–19 and a precursor and fragment mass tolerance of 20 ppm. Modifications specified were Carbamidomethyl @C fixed and Oxidation @M Variable common 1. The protein output was set to 1% FDR.

### Chick Biventer Cervicis Nerve-Muscle Preparation

Male chickens (aged 4–10 days) were killed by exsanguination following CO_2_ inhalation. Biventer cervicis nerve-muscle preparations were dissected and then mounted on wire tissue holders under 1 g resting tension in 5 ml organ baths. Tissues were maintained at 34°C, bubbled with 95% O_2_ and 5% CO_2_, in physiological salt solution of the following composition (mM): 118.4 NaCl, 4.7 KCl, 1.2 MgSO_4_, 1.2 KH_2_PO_4_, 2.5 CaCl_2_, 25 NaHCO_3_ and 11.1 glucose. Indirect twitches were evoked by stimulating the motor nerve (0.1 Hz; 0.2 ms) at supramaximal voltage (10–20 V), using a Grass S88 stimulator (Grass Instruments, Quincy, MA). Selective stimulation of the nerve was confirmed by the abolishment of twitches by the addition of d-tubocurarine (10 μM). Tissues were then repeatedly washed with physiological salt solution to restore twitch response to nerve stimulation. Contractile responses of the tissues to exogenous acetylcholine (ACh; 1 mM for 30 s), carbachol (CCh; 20 μM for 60 s) and KCl (40 mM for 30 s) were obtained in the absence of nerve stimulation. Nerve stimulation was then recommenced for at least 30 min before the addition of the toxin or antivenom.

To examine the ability of antivenom to neutralise α-elapitoxin-Oh3a (i.e., prevention study), tissues were equilibrated with antivenom for 10 min before the toxin was added. To determine the ability of antivenom to reverse α-elapitoxin-Oh3a induced neurotoxicity (i.e., reversal study), antivenom was added at t_90_ (i.e., time at which the initial twitch height was inhibited by 90%). To examine the reversible nature of the binding of α-elapitoxin-Oh3a to skeletal nAChR, organ baths were repeatedly washed for a period of 10 s every 5 min until any recovery of twitch responses had plateaued.

At the conclusion of each experiment, ACh, CCh and KCl were re-added as above. Twitch responses and responses to exogenous agonists were measured *via* a Grass FT03 force displacement transducer and recorded on a PowerLab system (ADInstruments Pty Ltd., Australia).

### Cumulative Concentration-Response Curves to Carbachol

In order to determine the potency (i.e., pA_2_ value) of α-elapitoxin-Oh3a, cumulative concentration–response curves to CCh (0.6–80 µM) were obtained by adding increasing concentrations of the agonist to unstimulated chick preparations without washing the preparation between each addition. After the maximum response was achieved, the tissue was repeatedly washed and allowed to recover for 30 min. Then α-elapitoxin-Oh3a (1–30 nM) or vehicle (milli-Q water) was allowed to equilibrate with the tissue for 60 min before the cumulative concentration–response curve to CCh was repeated in the presence of toxin or vehicle.

### Data Analysis

The quantity of α-elapitoxin-Oh3a in the whole venom was determined by measuring the area under the curve of the reverse-phase HPLC and ion-exchange chromatograms with the single peak representing α-elapitoxin-Oh3a being expressed as a percentage of the total area under the curve of whole venom.

Nerve-mediated twitch responses and responses to ACh (30 s), CCh (60 s) and KCl (30 s) were measured via a Grass FT03 force displacement transducer and recorded on PowerLab system (ADInstruments Pty Ltd., Australia). Post*-*venom/toxin responses were expressed as a percentage of their initial responses. An unpaired *t*-test or one-way analysis of variance (ANOVA) was used to compare the effect on twitch height of different pre-treatments. Comparison of responses to exogenous agonists before and after pre-treatment was made using a Student’s paired *t*-test. In order to determine the antagonist potency (i.e., pA_2_) of α-elapitoxin-Oh3a, the shifts in the cumulative concentration-response curves to CCh, in the absence or presence of α-elapitoxin-Oh3a, were analysed using the modified Lew Angus method. All ANOVAs were followed by a Bonferroni’s multiple comparison post-hoc test. Data presented are in the form of mean ± standard error of the mean (SEM) of n experiments. All data and statistical analyses were performed using PRISM 9.2.0 (GraphPad Software, San Diego, CA, United States, 2016). For all statistical tests, *p* < 0.05 was considered statistically significant.

## Results

### *In Vitro* Neurotoxicity of the Whole Venom

**FIGURE 1:**
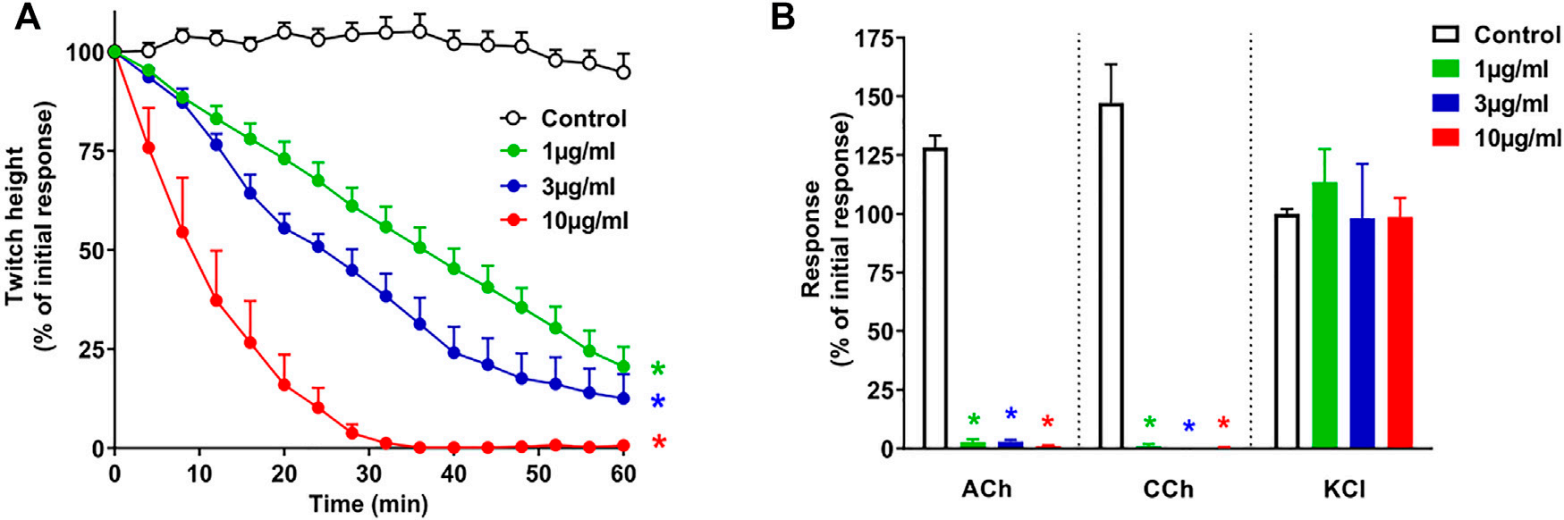
Concentration-dependent *in vitro* neurotoxicity of *O. hannah* venom (1–10 μg/ml) on **(A)** indirect twitches and **(B)** responses to exogenous agonists ACh (1 mM), CCh (20 µM) and KCl (40 mM) in the chick biventer cervicis nerve-muscle preparation. *p < 0.05, significantly different from **(A)** control at 60 min (one-way ANOVA; *n* = 4) or **(B)** pre-toxin response to same agonist (paired *t*-test, *n* = 4).

*O. hannah* venom (1–10 μg/ml) caused concentration-dependent inhibition of indirect twitches in the chick biventer cervicis nerve-muscle preparation (*n* = 4; *p* < 0.05, one-way ANOVA; Figure 1A). All concentrations of the venom also abolished contractile responses of tissues to exogenous ACh and CCh (*n* = 4; *p* < 0.05, paired *t*-test; Figure 1B), but not KCl. The reduction of responses to ACh and CCh, without affecting the responses to KCl, indicates a postsynaptic mode of action.

### Fractionation of Venom *via* Reverse-phase HPLC and Ion-Exchange Chromatography

**FIGURE 2:**
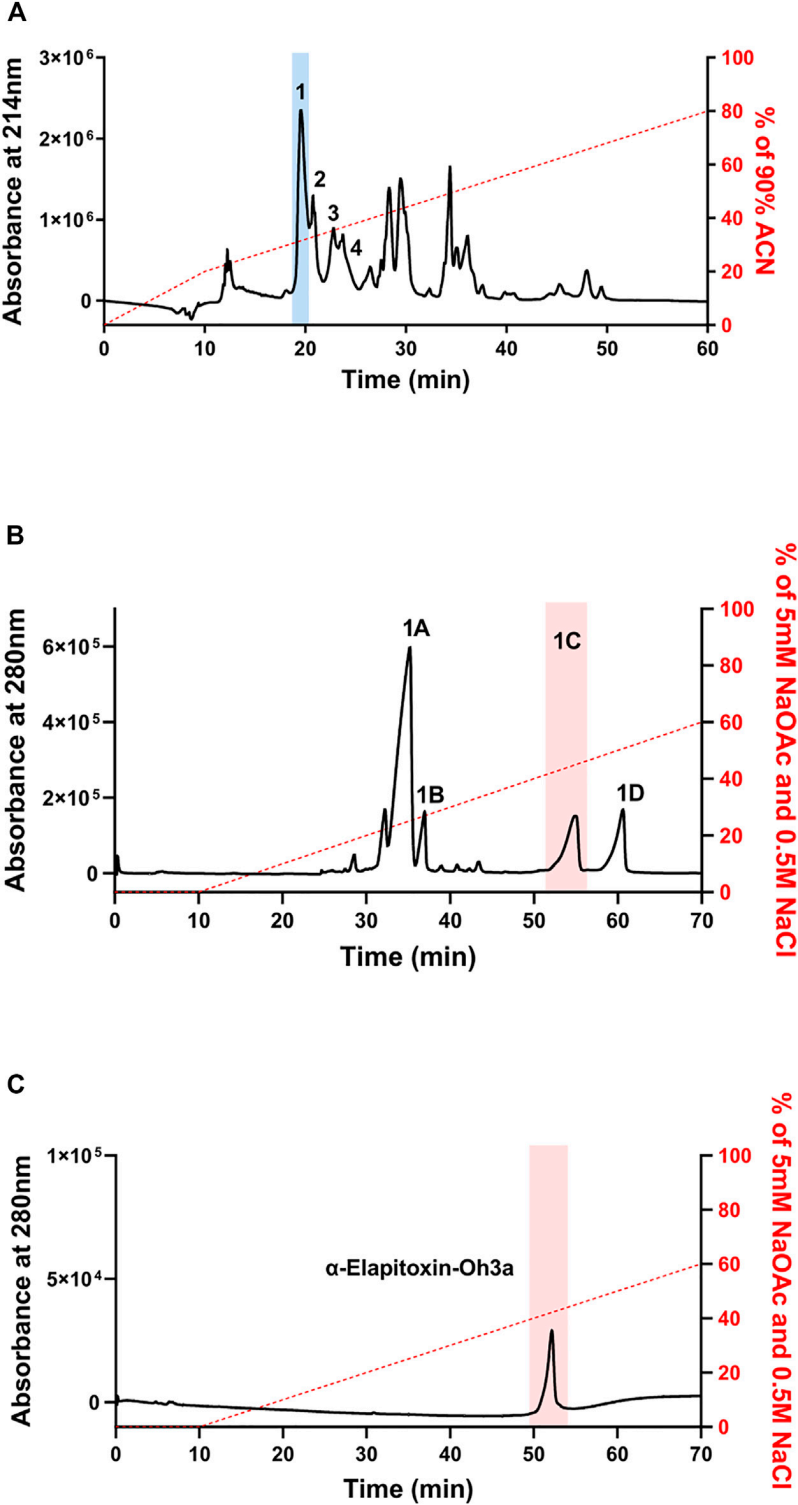
Chromatograms of *O. hannah* venom showing the fractionation steps to purify α-Elapitoxin-Oh3a. **(A)** *O. hannah* venom by reverse-phase HPLC on a Jupiter C18 semi-preparative column; **(B)** fractionation of peak 1 by ion-exchange chromatography on a Mono S strong cation exchange column; **(C)** further purification of peak 1C by ion-exchange chromatography on a Mono S strong cation exchange column.

α-Elapitoxin-Oh3a was isolated from *O. hannah* venom using reverse-phase HPLC followed by ion-exchange chromatography. Fractionation of whole venom by reverse-phase HPLC on a Jupiter C18 semi-preparative column yielded several major and minor peaks (Figure 2A). Initial screening of all fractions from reverse-phase HPLC, in the chick biventer nerve-muscle preparation, showed that peak 1, peak 2, peak 3 and peak 4, which eluted at 19, 20, 22 and 23 min, respectively, displayed postsynaptic neurotoxicity. Peak 1 was further purified using ion-exchange chromatography on a Mono S strong cation exchange column, producing several additional fractions (Figure 2B). Initial screening of these fractions, in the chick biventer nerve-muscle preparation, showed that peak 1C, which eluted at 52 min, displayed postsynaptic neurotoxicity. Fraction 1C, subsequently named α-elapitoxin-Oh3a, was subjected to further purification using ion-exchange chromatography, resulting in a clean peak (Figure 2C). α-Elapitoxin-Oh3a is estimated to constitute approximately 5% of the whole venom protein content. The neurotoxins within peak 2, peak 3 and peak 4 could not be further purified using the current chromatographic methods.

### Intact Protein Analysis With MALDI-TOF Mass Spectrometry

**FIGURE 3:**
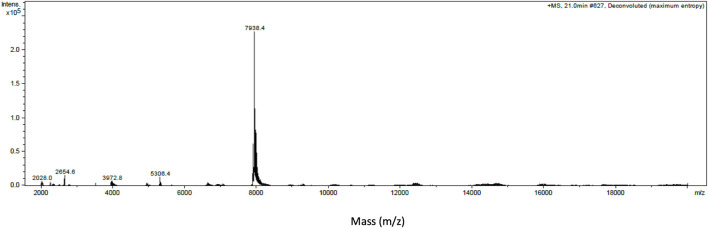
Intact protein MALDI-TOF chromatogram of α-Elapitoxin-Oh3a indicating an intact mass of 7,938.4 Da.

Intact protein analysis of α-elapitoxin-Oh3a using MALDI-TOF showed a single mass with a molecular weight of 7,938.4 Da (Figure 3).

### De Novo Protein Sequencing and Protein Identification

Enzyme digest using ESI-LCMS/MS generated the following full amino acid sequence of α-elapitoxin-Oh3a (72 amino acids) which was validated using Byonic (ProteinMetrics) V 3.1–19.

TKCYVTPDVKSETCPAGQDICY TETWCDAWCTSRGKRVNLGCAATCPIVKPGV EIKCCSTDNCNPFPTRKRP

**FIGURE 4:**
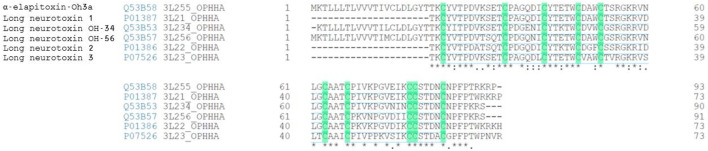
Sequence alignment (from BLAST search) of α-Elapitoxin-Oh3a with long-chain alpha neurotoxins from *O. hannah* venom. Highlighted amino acids are the 10 cysteine residues present in identical positions of all long-chain alpha neurotoxins that make the five disulfide bonds. Amino acids with (*) are fully conserved in all toxins, conserved amino acids with (.) are weakly similar properties group and amino acids with (:) are strongly similar properties group.

α-Elapitoxin-Oh3a showed 100% sequence identity with Long neurotoxin OH-55 (UniProtKB–Q53B58) and was assigned the name “α-elapitoxin-Oh3a” based on the rational nomenclature for naming peptide toxins from spiders and other venomous animals suggested by King et al. (2008). α-Elapitoxin-Oh3a showed more than 80% sequence homology with the long-chain α-neurotoxins, long neurotoxin 1, long neurotoxin 2, long neurotoxin 3, long neurotoxin OH-34, long neurotoxin OH-56 isolated from the venom of *O. hannah* (Figure 4).

### *In Vitro Neurotoxicity of* α-Elapitoxin-Oh3a

#### Concentration-Dependent Inhibition of Twitches and Exogenous Agonists Responses

**FIGURE 5:**
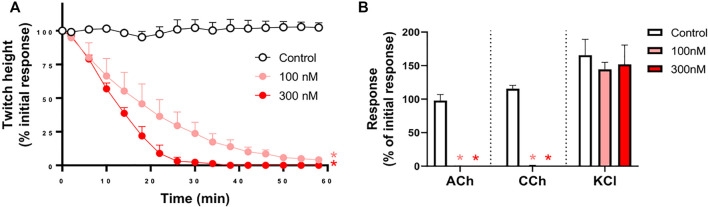
The concentration-dependent *in vitro* neurotoxic effects of α-elapitoxin-Oh3a (100–300 nm) on **(A)** indirect twitches and **(B)** responses to exogenous agonists ACh (1 mM), CCh (20 µM) and KCl (40 mM) in the chick biventer cervicis nerve-muscle preparation. *p < 0.05, significantly different from **(A)** control at 60 min (one-way ANOVA; *n* = 4) or **(B)** pre-toxin response to same agonist (paired *t*-test, *n* = 4).

α-Elapitoxin-Oh3a (100–300 nM) caused concentration-dependent inhibition of indirect twitches in the chick biventer cervicis nerve-muscle preparation (*n* = 4; *p* < 0.05, one-way ANOVA; Figure 5A). Both concentrations of the toxin inhibited responses of tissues to exogenous ACh and CCh (*n* = 4; *p* < 0.05, paired *t*-test; Figure 5B), but not KCl. The reduction of responses to ACh and CCh, without affecting the responses to KCl, indicates a postsynaptic mode of action.

#### *In Vitro* Neurotoxicity Antivenom Prevention Study

**FIGURE 6:**
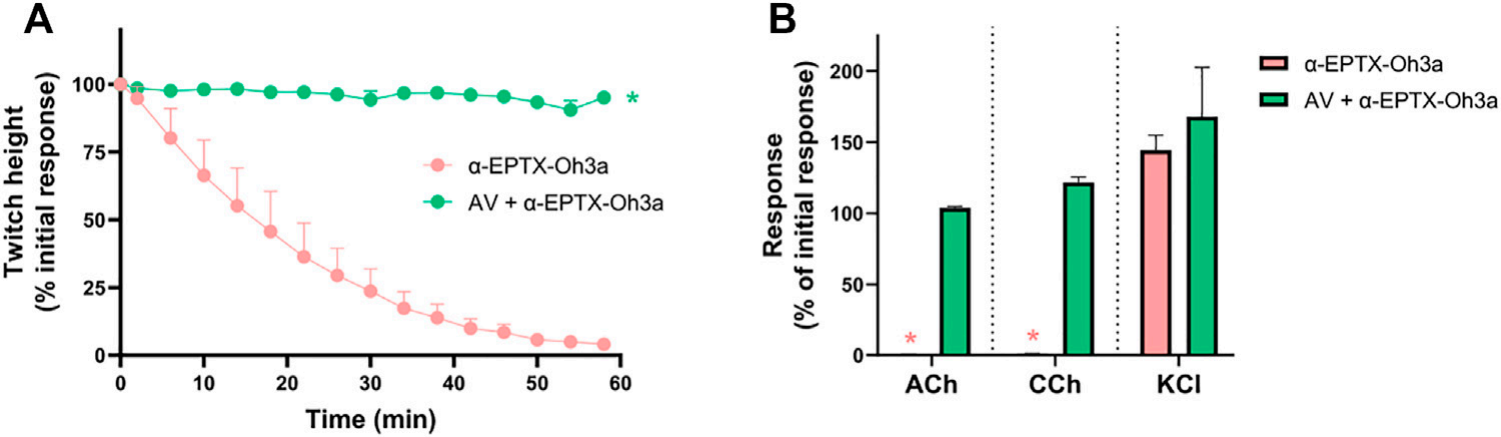
The effect of α-elapitoxin-Oh3a (100 nM) in the absence and presence of Thai Red Cross Society King Cobra antivenom (AV; at the recommended concentration; 1 ml/0.8 mg) on **(A)** indirect twitches and **(B)** responses to exogenous agonists ACh (1 mM), CCh (20 µM) and KCl (40 mM) in the chick biventer cervicis nerve-muscle preparation. *p < 0.05, significantly different from **(A)** α-elapitoxin-Oh3a alone at 60 min (unpaired *t*-test; *n* = 4) or **(B)** pre-toxin response to same agonist (paired *t*-test, *n* = 4).

The prior incubation of tissues with Thai Red Cross Society King Cobra antivenom (1 ml/0.8 mg) prevented the inhibition of indirect twitches by α-elapitoxin-Oh3a (100 nM; *n* = 4; *p* < 0.05, unpaired *t*-test; Figure 6A) and prevented the reduction of contractile responses to ACh and CCh (*n* = 4; *p* < 0.05, paired *t*-test; Figure 6B).

#### *In Vitro* Neurotoxicity Antivenom Reversal and Washing Study

**FIGURE 7:**
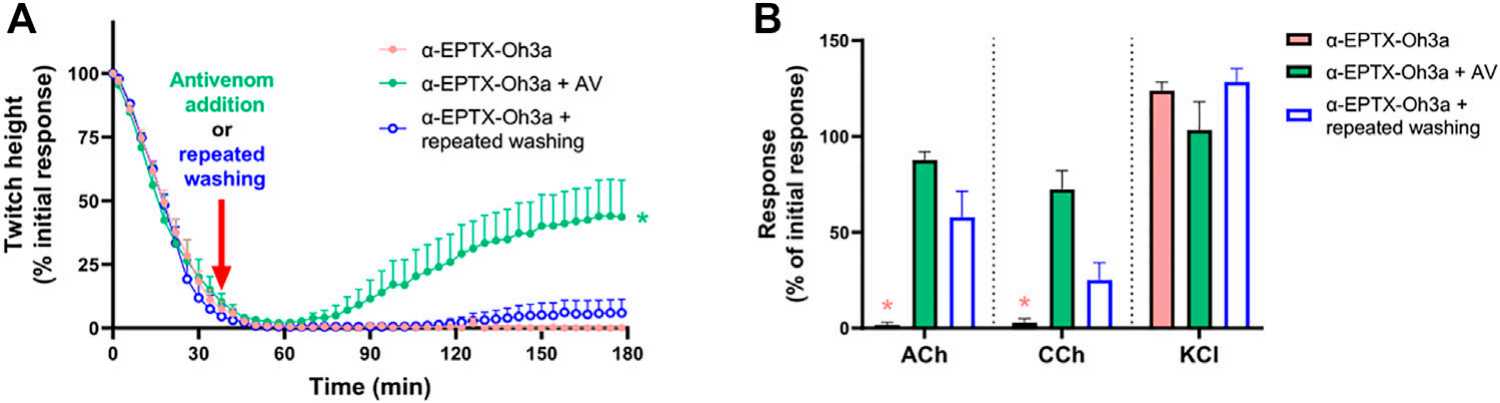
The effect of α-elapitoxin-Oh3a (100 nM) in the absence and presence of Thai Red Cross Society King Cobra antivenom (AV; at the recommended concentration; 1 ml/0.8 mg) or repeated washing of the tissue at the t_90_ time point on **(A)** indirect twitches and **(B)** responses to exogenous agonists ACh (1 mM), CCh (20 µM) and KCl (40 mM) in the chick biventer cervicis nerve-muscle preparation. *p < 0.05, significantly different from **(A)** α-elapitoxin-Oh3a alone at 180 min (one-way ANOVA; *n* = 4) or **(B)** pre-toxin response to same agonist (paired *t*-test, *n* = 4).

The addition of Thai Red Cross Society King Cobra antivenom (1 ml/0.8 mg), at the t_90_ time point following the effect of α-elapitoxin-Oh3a (100 nM), partially restored the twitch responses to 42.0 ± 14.3% (*n* = 4; *p* < 0.05, one-way ANOVA; Figure 7A) of the pre-toxin twitch height by the 180 min time point. The toxin-induced reduction of responses to ACh and CCh was also significantly prevented by the delayed addition of antivenom (*n* = 4; *p* < 0.05, paired *t*-test; Figure 7B).

Repeatedly washing the tissue for 10 s every 5 min, commencing at the t_90_ time point following addition of α-elapitoxin-Oh3a (100 nM), only caused a slight recovery of the twitch responses to 6.4 ± 5.6% (*n* = 4; Figure 7A). Repeated washing also partially reversed the inhibition of responses to ACh and CCh (*n* = 4; *p <* 0.05, paired *t*-test; Figure 7B).

### Carbachol Cumulative Concentration-Response Curves

**FIGURE 8:**
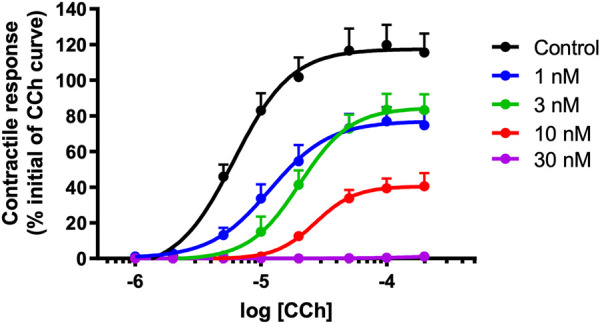
Concentration-dependent effects of α-elapitoxin-Oh3a (1–30 nM; *n* = 5) on cumulative concentration-response curves to carbachol (CCh) in the unstimulated chick biventer cervicis nerve-muscle preparation.

α-Elapitoxin-Oh3a (1–30 nM) caused a concentration-dependent non-parallel shift of the cumulative concentration-response curve to CCh, with a concentration-dependent reduction of the maximum response in unstimulated chick biventer cervicis nerve-muscle preparations (Figure 8). This indicates that α-elapitoxin-Oh3a is pseudo-irreversible antagonist at the skeletal nAChR. Using the modified Lew and Angus method, the pA_2_ value of α-elapitoxin-Oh3a was calculated to be 8.99 (95% c.l.: 8.00–9.90).

## Discussion

In this study, we examined the *in vitro* neurotoxicity of *O. hannah* venom, and isolated and characterized a long-chain postsynaptic α-neurotoxin, α-elapitoxin-Oh3a. The *in vitro* neurotoxicity of α-elapitoxin-Oh3a was neutralized, and partially reversed, by Thai Red Cross Society King Cobra antivenom. Administration of antivenom provided more recovery than repeated washing of the tissue. α-Elapitoxin-Oh3a also demonstrated irreversible or “pseudo-irreversible” antagonism at avian skeletal nAChR.

*O. hannah* venom demonstrated postsynaptic neurotoxicity in the indirectly stimulated chick biventer nerve-muscle preparation as indicated by the concentration-dependent inhibition of indirect twitches and reduction in contractile responses to external nicotinic agonists. This observation is consistent with proteomic reports suggesting a large abundance of postsynaptic neurotoxins in the venom (Petras et al., 2015; Tan et al., 2015).

α-Elapitoxin-Oh3a was isolated using reverse-phase HPLC and ion-exchange chromatography, and shown to constitute approximately 5% of *O. hannah* venom. With a molecular mass of 7,938.4 Da, and consisting of 72 amino acids, α-elapitoxin-Oh3a exhibits traits consistent with those of long-chain postsynaptic neurotoxins (Barber et al., 2013). Further, α-elapitoxin-Oh3a was calculated to have a pA_2_ value of 8.99, indicating a high potency in terms of its antagonist activity at the nAChR. For comparison, the pA_2_ of α-bungarotoxin, a potent long-chain α-neurotoxin, determined using the same methodology as the current study, was calculated to be 8.71 (Wickramaratna et al., 2004). Our experiments also suggest that the antagonism of α-elapitoxin-Oh3a is irreversible or pseudo-irreversible. All the above are suggestive of α-elapitoxin-Oh3a being a typical long-chain α-neurotoxin (Barber et al., 2013).

Additionally, we identified three fractions from reverse-phase HPLC which displayed postsynaptic neurotoxicity, but the α-neurotoxins within these fractions could not be further purified using ion-exchange chromatography. The presence of multiple α-neurotoxins in *O. hannah* venom from Indonesia, has also been previously indicated by proteomic analysis (Petras et al., 2015), with approximately 37% of the venom protein identified as postsynaptic neurotoxins. α-Elapitoxin-Oh3a showed 100% sequence identity with Long neurotoxin OH-55, which was also reported by the aforementioned study to constitute approximately 12% of the venom, slightly more than the relative abundance suggested in the current study.

Long neurotoxin OH-55 has also been isolated from *O. hannah* venom by He et al. (2004). In this previous study, a 24 h LD_50_ assay of the isolated α-neurotoxins from *O. hannah* venom in mice showed Long neurotoxin OH-55 to have a LD_50_ of 120 ng/g (i.p.), likely due to a high potency on the mouse (rodent) nAChR. Unlike short-chain α-neurotoxins, long-chain α-neurotoxins do not show a difference in the potency and reversibility between human and rodent nAChR. Therefore, they are likely to be more clinically important and capable of causing neuromuscular paralysis in humans (Silva et al., 2018). Hence, it could be assumed that α-elapitoxin-Oh3a, which constitutes 5–12% of the whole venom, would be of high clinical importance in causing neuromuscular paralysis in envenomed humans.

Thai Red Cross Society King Cobra antivenom was able to fully prevent the neurotoxic effects of α-elapitoxin-Oh3a, indicating the presence of antibody fragments against the toxin. Further, the antivenom was able to partially restore (∼40%) the indirect twitches of the chick biventer preparation after they were fully inhibited by the toxin. The continuous physical removal of α-elapitoxin-Oh3a, by repeated washing of the preparation, did not result in any significant reversal of the neurotoxic effect of the toxin on indirect twitches although there was partial restoration of the responses to exogenous agonists. Since the constant removal of toxin from the surrounding physiological environment is unable to effectively reverse the neuromuscular block, this suggests that the role of antivenom may not be limited to antibodies binding to epitopes at the pharmacological site of unbound toxins, preventing their antagonism at nAChRs, but antibodies may also bind to epitopes more distant from the pharmacological site of nAChR-bound toxins, triggering conformational changes in the toxin that would reduce its affinity for nAChRs (Silva and Isbister, 2020). This was previously demonstrated by commercially used antivenoms and high-affinity monoclonal antibodies which could accelerate the detachment of *Naja nigricollis* toxin α from nAChRs (Boulain and Ménez, 1982; Boulain et al., 1985; Gatineau et al., 1988). In contrast to α-elapitoxin-Oh3a, toxin α is a short-chain postsynaptic neurotoxin, which generally have a lower potency and higher reversibility at nAChRs, and likely to be of lesser clinical importance (Silva et al., 2018). The important observation made in this study is that antivenom can reverse the clinically important long-chain α-neurotoxin-mediated neuromuscular paralysis.

In conclusion, we have shown that α-Elapitoxin-Oh3a (7,938 Da), a long-chain a-neurotoxin from *O. hannah* venom, is a highly potent pseudo-irreversible antagonist at nAChR in avian skeletal muscle. The neurotoxic effects of α-Elapitoxin-Oh3a are prevented, and partially reversed, by Thai Red Cross Society King Cobra antivenom. α-Elapitoxin-Oh3a is likely to be a key contributor the neuromuscular paralysis that can result by envenoming by the King Cobra.

## Data Availability

The protein sequence data reported in this paper is available in the UniProt Knowledgebase under the accession number Q53B58.
